# *Ex vivo* Sealing Performance of a Sutureless Dual-Component Connector for Coronary Bypass

**DOI:** 10.1093/icvts/ivaf270

**Published:** 2025-11-06

**Authors:** Monica Gianoli, Harmen M Wassink, Leonard J van Schelven, Paul F Gründeman, Willem J L Suyker

**Affiliations:** Cardiothoracic Surgery, Heart and Lungs Division, UMC Utrecht, The Netherlands; Medical Technology and Clinical Physics, UMC Utrecht, The Netherlands; Medical Technology and Clinical Physics, UMC Utrecht, The Netherlands; Cardiothoracic Surgery, Heart and Lungs Division, UMC Utrecht, The Netherlands; Cardiothoracic Surgery, Heart and Lungs Division, UMC Utrecht, The Netherlands

**Keywords:** anastomotic connector device, coronary revascularization, vascular connector technology

## Abstract

Sutureless coronary connectors may enable minimally invasive bypass surgery, but consistent sealing in human vessels remains a key challenge. This *ex vivo* study evaluates the hydrostatic performance of a titanium connector that employs a novel sealing principle: tissue-to-metal coaptation enabling stepwise distal anastomosis construction. Thirteen anastomoses were created in porcine and human cadaveric vessels using a standardized three-step deployment protocol. Target sites included internal mammary arteries, saphenous veins, and coronary arteries, selected for suitability for hand-suturing. Sealing was assessed by hydrostatic pressure testing up to 300 mmHg. Key anatomical parameters and procedural outcomes were recorded. Twelve of 13 anastomoses were completed without leakage up to 300 mmHg; one case exhibited minor oozing at 260 mmHg. The connector accommodated vessels down to 1.3 mm in diameter and up to 0.8 mm wall thickness. One misdeployment due to an oversized incision was corrected intraoperatively without compromising the hydrostatic result. Visual inspection confirmed wide, unobstructed anastomotic orifices. This *ex vivo* evaluation demonstrates that the connector achieved consistent hydrostatic sealing through bare-metal vessel coaptation, even in small-diameter human coronary tissue. These findings support further *in vivo* validation towards minimally invasive coronary bypass applications.

## INTRODUCTION

Minimally invasive and endoscopic coronary artery bypass grafting (CABG) is gaining renewed momentum. Patients increasingly expect faster recovery, hospitals demand greater efficiency, and surgeons are embracing robotic platforms. Industry investment is accelerating. Yet one critical barrier persists: the distal anastomosis.^1^

Since the discontinuation of the C-Port system (Cardica Inc., Redwood, CA, USA), no anastomotic connector has been available to facilitate totally endoscopic CABG (TECAB). While C-Port demonstrated that mechanical anastomosis could achieve satisfactory patency, it struggled to gain clinical adoption due to procedural complexity, anatomical limitations, and high cost. Patency alone is not enough—ease of use, anatomical versatility, and cost-effectiveness are equally critical.^2^

Other investigational devices fell short in terms of patency or usability and did not progress to routine clinical use. Together, these experiences demonstrate that long-term patency alone is insufficient; a successful connector must also provide versatility, procedural simplicity, and cost-effectiveness.

The clinical need for such a connector remains unmet, particularly to enable truly minimally invasive and endoscopic CABG. We therefore developed a dual-component titanium connector (Octocon) designed to meet these requirements. The system integrates controlled expansion and micro-stapling with a click-lock coupling, enabling stepwise deployment on the graft and target vessel. Its oval orifice (∼7 mm^2^) with 12 staples mimics hand-sewn geometry while limiting blood-exposed non-intimal surface (BENIS) (∼3 mm).

A key novelty is the sealing principle: direct tissue-to-metal vessel wall coaptation, providing immediate hemostasis before biological integration. To our knowledge, this is the first application of such a strategy in a coronary connector. The present study evaluates the immediate sealing performance of Octocon by *ex vivo* hydrostatic testing in porcine and human coronary vessels.

## METHODS

We performed *ex vivo* testing of the Octocon prototype using porcine and human cadaveric hearts. Donor vessels included porcine internal mammary arteries, human internal mammary arteries, and human saphenous vein graft segments. IRB/EC approval and informed consent were not required.

Target vessels included porcine internal mammary arteries, human left internal mammary arteries (LIMA), human saphenous vein grafts (SVG), and human coronary arteries (LAD and diagonals), some of which exhibited visible atherosclerosis. In all cases, the anastomotic sites were chosen to represent locations that would be suitable for conventional hand-suturing. All grafts and target vessels were prepared using standard protocols. Diameters were assessed under low static pressure (50–100 mmHg). Wall thicknesses were measured with a micrometer. Longitudinal incisions (∼3.0 mm) were made using visual alignment with a metal template to match the device’s expandable head.

The Octocon deployment followed a standardized three-step process: (1) insertion and expansion of the first connector half in the bypass graft; (2) insertion and expansion of the second connector half in the coronary artery; and (3) precise alignment and click-joining of the two halves, producing a side-to-side anastomosis that was converted to an end-to-side geometry by closing the graft’s free end. Device expansion was used to position the vessel walls predictably around the applicator, ensuring the vessel tissue was consistently presented to the staples before penetration (**Figure S1**).

Hydrostatic testing was performed by connecting each vessel segment to a saline-filled vertical column and measuring pressures via a calibrated manometer. Each anastomosis was pressurized to 200-300 mmHg and held for several minutes. Subsequently, vessels were opened longitudinally to inspect anastomotic geometry and patency.

## STATISTICAL ANALYSIS

Due to the limited sample size, formal statistical analysis was not performed. Descriptive statistics, including means, ranges, and frequencies, were calculated to summarize the findings.

## RESULTS

During 3 experimental sessions, 13 consecutive Octocon anastomoses were performed across porcine and human cadaveric models (**Table 1**). The connector’s expandable head required a ∼3.0 mm incision for insertion; if too short, the head could not enter, while over-enlargement risked loose fit (**Video 1**). In one case, an oversized incision was successfully corrected by placing a 7–0 Prolene suture to adjust the length and allowed a correct deployment.

**Table 1.** Anastomotic Integrity and Pressure Testing Result

	Anastomosis number	1	2	3	4	5	6	7	8	9	10	11	12	13
	Vessels connected	Human SVG				Porcine Lima	Human Lima					Human SVG		
		Porcine LAD					Human LAD			Human Diagonal	Human LAD			
**Graft**	OD, mm	4.0	4.0	4.0	4.0	3.6	3.0	2.8	2.6	3.0	3.0	4.5	4.5	4.5
	Incision length (mm)	3.2	3.1	3.0	3.1	2.9	2.8	3.0	3.0	3.0	3.0	3.0	3.0	3.1
**Coronary**	OD (mm)	2.6	3.0	2.5	1.9	2.6	3.2	2.3	3.8	2.0	3.8	2.5	2.6	2.7
	Incision length (mm)	3.0	2.8	3.0	3.0	3.0	3.1	3.0	3.1	3.1	3.5	2.9	2.9	3.0
	ID (mm)	-	-	-	1.4	-	-	1.5	3.0	1.3	-	-	-	-
	Wall thickness (mm)	-	-	-	-	-	-	-	-	0.2	0.8	-	-	-
	Remarks	-	-	-	Small	-	-	Small	Thick	Small, thin	Thick	-	-	-
**Pressure test**	Leakage at 200 mmHg	none	n.a.^a^	None	None	None	None	None	None	None	None	None	None	None
	Maximal pressure tested mmHg	300	n.a.^a^	300	300	300	300	300	300	230	300	300	300	250
	Remarks		Damaged connector										Oozing above 260 mmHg	

Abbreviations: ID, inside diameter; LAD, left anterior descending artery; Lima, left internal mammary artery; mm, millimeters; mmHg, millimeters of mercury; n.a., not available; OD, outside diameter; SVG, saphenous vein graft.

a This connection was excluded from the analysis due to inappropriate maneuvers on the connector plates, rendering the result unrepresentative.

Twelve completed anastomoses underwent static hydrostatic testing; one was excluded due to improper handling of the connector plates during deployment. Of the remaining 12, all reached 200 mmHg without leakage or failure and nine withstood 300 mmHg. Two reached maximum pressures of 230 mmHg and 250 mmHg, respectively, due to technical limitations of the setup, but showed no leakage. One anastomosis displayed minor oozing at 260 mmHg. All connections performed with human LIMA and visible atherosclerotic coronary vessels remained fluid-tight at physiologic and supraphysiologic pressures up to 300 mmHg.

**Figure 1.**
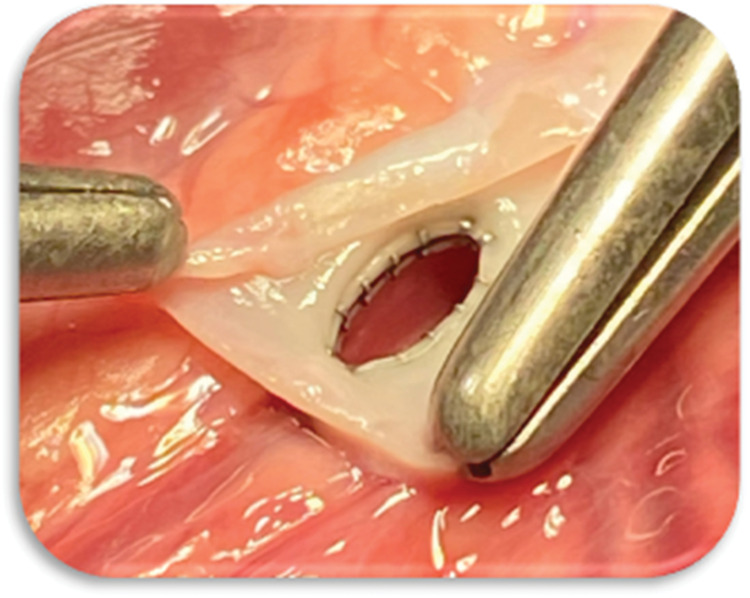
The Octocon Anastomosis Mimicking Hand-Sewn Geometry. Zoom in of a Porcine “Cut Open” IMA to LAD Anastomosis. Abbreviations: LAD: Left Anterior Descending Artery; IMA: Internal Mammary Artery.

Visual inspection after vessel opening confirmed fully patent, unobstructed anastomotic orifices in all valid cases. The connector halves consistently achieved secure wall apposition, and the stepwise deployment enabled reproducible alignment, coaptation, and subsequent fixation of the connector components. No instances of device displacement or misalignment were observed. All wall coaptation and connector integration were consistent across samples (**Figure 1**).

Overall, the Octocon system demonstrated adaptability to a range of vessel sizes (≥1.3 mm inside diameter) and varying wall thicknesses (range 0.2-0.8 mm), successfully creating patent, leak-free anastomoses under high static pressures in a consistent and reproducible manner.

## DISCUSSION

This study demonstrates consistent hydrostatic sealing of tissue to bare titanium in *ex vivo* porcine and human coronary arteries, at anatomical sites suitable for conventional hand-suturing. This novel vessel wall coaptation strategy marks a critical step towards converting anastomosis construction into a stepwise procedure.

At first glance, the approach may appear counterintuitive. Unlike traditional suturing that creates a single tissue-to-tissue interface, Octocon introduces 3: tissue-to-metal at both graft and target vessel, and a central metal-to-metal seal between connector halves. The latter should inherently be leakproof by design. The focus, therefore, lies on securing hemostasis at the tissue-to-metal interfaces.

This was achieved through two mechanisms: (1) radial expansion to evenly pre-stretch the vessel rims; and (2) deployment of twelve titanium micro-staples, spaced to replicate skilled surgical suturing, to fix the stretched vessel wall firmly against the metal. Staple geometry was carefully optimized to accommodate a fourfold variation in wall thickness, whether due to vessel size or local irregularities.

Hydrostatic testing up to 300 mmHg confirmed consistent sealing. While the addition of a soft, compliant substance like PTFE might theoretically enhance sealing, it proved unnecessary. Repositioning during deployment did not compromise hydrostatic performance—an essential feature for surgical workflows. Although the test setup was non-pulsatile, we consider it clinically relevant for evaluating hemostasis, as the absence of coagulation and the lower viscosity of water compared to blood present a more stringent sealing challenge.

Previously conducted destructive traction testing showed that staple pullout force exceeds vessel wall strength, confirming that failure occurs via tissue tear-out rather than staple deformation—behaviour consistent with surgical sutures. At the pressures tested, this failure mode did not occur. The click-lock mechanism should include intrinsic redundancy to ensure permanent fixation after deployment, irrespective of pressure or unexpected traction.

The user-friendliness of Octocon’s stepwise deployment was demonstrated earlier in robotic, totally endoscopic CABG (TECAB)—arguably one of the most demanding surgical settings.^3^ We consider early evaluation of usability a critical part of device development. Regarding the key determinant of long-term patency, Octocon was designed to replicate critical features of hand-sewn anastomoses while offering simple, controlled deployment. Our previously published meta-analysis highlighted the importance of adequate anastomotic orifice area and low BENIS.^4^ Octocon’s ∼7 mm^2^ orifice and ∼3 mm^2^ BENIS are expected to support favourable long-term outcomes.

Chronic animal data from our previous S2 microstapler demonstrated that tissue stretching in combination with microstapling—despite a non-compliant metal ring—resulted in unimpaired anastomosis healing and excellent patency.^5^^,^^6^ A chronic animal evaluation of Octocon will follow as the next step towards first-in-human application.

## CONCLUSION

This *ex vivo* study demonstrates that the Octocon connector can achieve reproducible metal-to-vessel sealing in porcine and human coronary tissues. Its hydrostatic performance and anatomical applicability suggest potential for use in CABG. These findings support progression to *in vivo* studies as the next step towards clinical translation.

## Supplementary Material

ivaf270_Supplementary_Data

## Data Availability

The data that support the findings of this study are available from the corresponding author upon reasonable request.
