# Supplementary material for: Ex vivo Sealing Performance of a Sutureless Dual-Component Connector for Coronary Bypass
Source: Interdiscip Cardiovasc Thorac Surg. 2025 Nov 6;40(11):ivaf270. doi: 10.1093/icvts/ivaf270 (PMC12709125; doi:10.1093/icvts/ivaf270)
Supplement: ivaf270_Supplementary_Data [file ivaf270_Supplementary_Data.zip › Supplemental.docx]

**Supplemental**

**Figure legend:**

**Figure S1:** Octocon deployment: A: Stapling head insertion into vessel. B: Stapling head expansion. C: Connector deployment. D: Both connectors placed. E: Click connectors together.
