# Supplementary figures and images for: Ex vivo Sealing Performance of a Sutureless Dual-Component Connector for Coronary Bypass
Source: Interdiscip Cardiovasc Thorac Surg. 2025 Nov 6;40(11):ivaf270. doi: 10.1093/icvts/ivaf270 (PMC12709125; doi:10.1093/icvts/ivaf270)

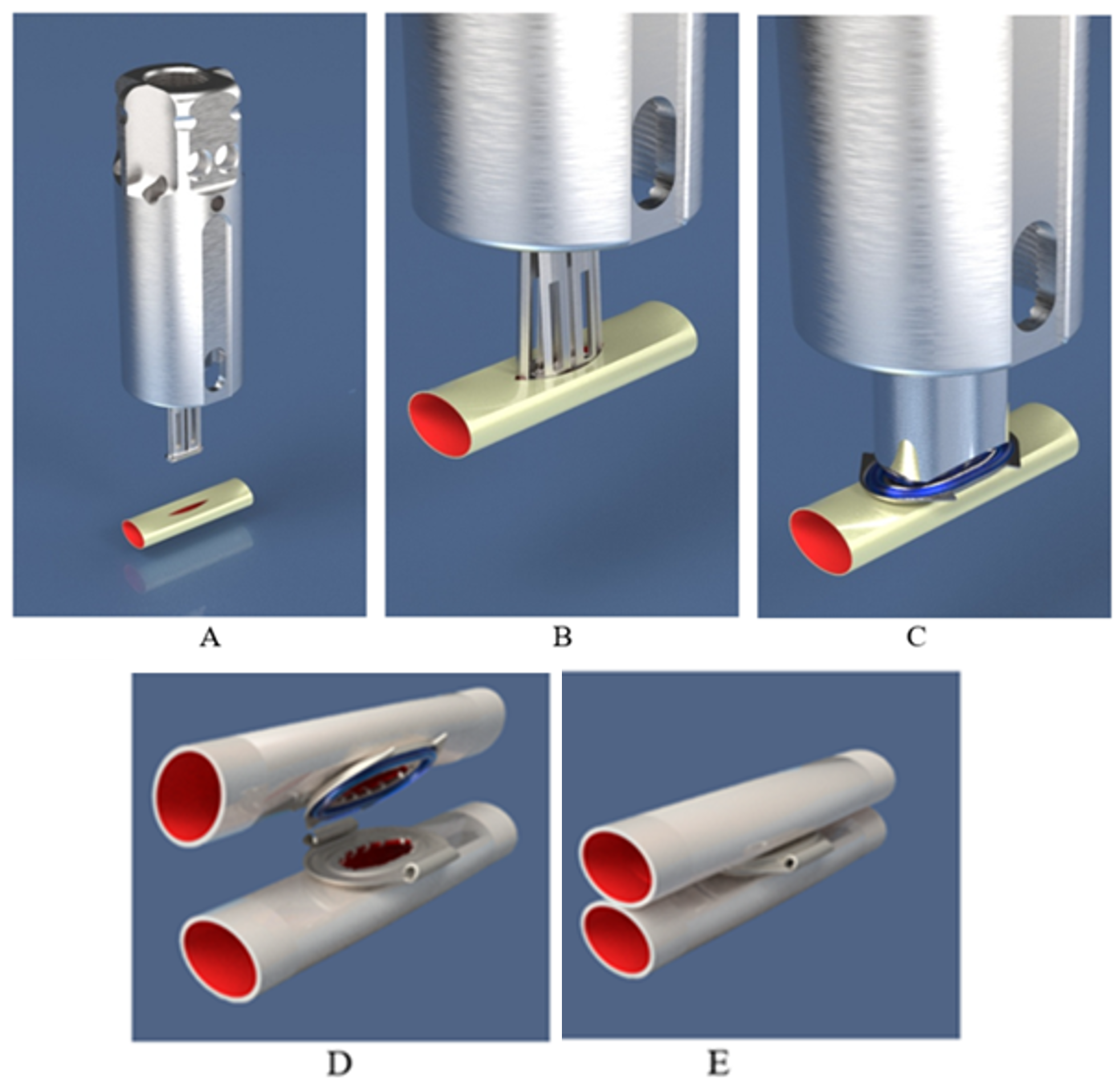

Supplement: ivaf270_Supplementary_Data [file ivaf270_Supplementary_Data.zip › Supplemental Figure S1.png]
